# Spatial and seasonal variability of the mass concentration and chemical composition of PM_2.5_ in Poland

**DOI:** 10.1007/s11869-013-0222-y

**Published:** 2013-12-08

**Authors:** Wioletta Rogula-Kozłowska, Krzysztof Klejnowski, Patrycja Rogula-Kopiec, Leszek Ośródka, Ewa Krajny, Barbara Błaszczak, Barbara Mathews

**Affiliations:** 1 Institute of Environmental Engineering, Polish Academy of Sciences, 34 M. Skłodowska-Curie St., 41-819 Zabrze, Poland; 2 Monitoring and Modeling of Air Pollution Department, Institute of Meteorology and Water Management–National Research Institute, 10 Bratków St., 40-045 Katowice, Poland

**Keywords:** Fine particulate matter, Secondary inorganic aerosol, Elemental carbon, Benzo(*a*)pyrene, Toxic metals, Chemical mass closure

## Abstract

The seasonal changes in ambient mass concentrations and chemical composition of fine particulate matter (PM_2.5_) were investigated in three locations in Poland. The analyses included PM_2.5_-bound hazardous benzo(*a*)pyrene (BaP), As, Ni, Cd, and Pb. The samples of PM_2.5_ were collected daily in Katowice (southern Poland, urban background site), Gdańsk, and Diabla Góra (northern Poland, urban and regional background sites, respectively) during 1-year-long campaign in 2010. Based on monthly ambient concentrations of PM_2.5_-bound carbon (organic and elemental), water-soluble ions (Na^+^, NH_4_^+^, K^+^, Mg^2+^, Ca^2+^, Cl^−^, NO_3_^−^, SO_4_^2−^), and elements As, Ni, Cd, Pb, Ti, Al, Fe, the chemical mass closure of PM_2.5_ was checked for each of the four seasons of the year and for the heating and non-heating periods at each site. Also, the annual concentrations of PM_2.5_ were determined and the annual PM_2.5_ mass closure checked. At each measuring point, the PM_2.5_ concentrations were high compared to its Polish yearly permissible value, 25 μg/m^3^, and its concentrations elsewhere in Europe. The highest annual PM_2.5_ concentration, 43 μg/m^3^, occurred in Katowice; it was twice the annual PM_2.5_ concentration in Gdańsk, and thrice the one in Diabla Góra. The high annual averages were due to very high monthly concentrations in the heating period, which were highest in the winter. PM_2.5_ consisted mainly of carbonaceous matter (elemental carbon (EC) + organic matter (OM), the sum of elemental carbon, EC, and organic matter, OM; its annual mass contributions to PM_2.5_ were 43, 31, and 33 % in Katowice, Gdansk, and Diabla Góra, respectively), secondary inorganic aerosol (SIA), the Na_Cl group, and crustal matter (CM)—in the decreasing order of their yearly mass contributions to PM_2.5_. OM, EC, SIA, Na_Cl, and CM accounted for almost 81 % of the PM_2.5_ mass in Katowice, 74 % in Gdańsk, and 90 % in Diabla Góra. The annual average toxic metal contribution to the PM_2.5_ mass was not greater than 0.2 % at each site. In Katowice and Gdańsk, the yearly ambient BaP concentrations were high (15.4 and 3.2 ng/m^3^, respectively); in rural Diabla Góra, the concentrations of BaP were almost equal to 1 ng/m^3^, the Polish BaP annual limit. The great seasonal fluctuations of the shares of the component groups in PM_2.5_ and of the concentrations of PM_2.5_ and its components are due to the seasonal fluctuations of the emissions of PM and its precursors from hard and brown coal combustion for energy production, growing in a heating season, reaching maximum in winter, and decreasing in a non-heating period. In Gdańsk, northern Poland, especially in the spring and autumn, sea spray might have affected the chemical composition of PM_2.5_. The greatest hazard from PM_2.5_ occurs in Katowice, southern Poland, in winter, when very high concentrations of PM_2.5_ and PM_2.5_-related carbonaceous matter, including BaP, are maintained by poor natural ventilation in cities, weather conditions, and the highest level of industrialization in Poland. In less industrialized northern Poland, where the aeration in cities is better and rather gaseous than solid fuels are used, the health hazard from ambient PM_2.5_ is much lower.

## Introduction

The threat posed by ambient particulate matter (PM) to the health is one of the greatest, and yet growing, air pollution concerns of great cities worldwide (Molina and Molina 2004; Kong et al. 2012). High concentrations of PM increase the risk of serious diseases in humans (Pope and Dockery 2006; Kappos 2011). The risk arises from the combined effects of the chemical composition and the size of PM particles. The finer a particle is, the greater variety and amount of toxic metals, polycyclic aromatic hydrocarbons, etc. it comprises and the deeper it penetrates into the respiratory tract (Englert 2004; Ostro et al. 2007; Cho et al. 2009). The fine particles (aerodynamic diameters up to 2.5 μm, PM_2.5_) can reach the deepest regions of lungs and get into blood; their adverse effects are much more serious than those of the coarse particles (diameter between 2.5 and 10 μm, Zhang et al. 2011; Baxter et al. 2013).

A great number of studies report the PM_2.5_ mass concentration and chemical composition as functions of the particle size at a variety of types of European stations (Table 1).

**Table 1:** Mass concentrations of fine PM (in microgram per cubic) and its grouped element content (in percent) at selected urban, suburban, and rural sites in Europe (EC + OM—sum of EC-elemental carbon and OM-organic matter, SIA—secondary inorganic aerosol, CM/MM—crustal/mineral matter, SS/Na_Cl—sea salt/Na_Cl, TE/OE—trace/other elements)

City (country), site type, averaging period; PM fraction (data source)	PM (μg/m^3^)	EC + OM (%)	SIA (%)	CM/MM (%)	SS/Na_Cl (%)	TE/OE (%)
London (UK), background, year; PM_2.5_ (Harrison et al. 2004)	14.35	39.5	35.9	12.1^a^	2.1	–
Streithofen (Austria), rural background, year; PM_2.5_ (Puxbaum et al. 2004)	18.1	36	46	–	1	<1
Montseny (Spain), rural background, year; PM_2.5_ (Rodriguez et al. 2004)	13.6	27	38	10	1	<1
Thüringer Wald (Germany), rural background, October; PM_0.42–1.2_ (Gnauk et al. 2005)	9.6	24	46	–	–	–
Basel (Switzerland), suburban, year; PM_2.5_ (Hüeglin et al. 2005)	18.9	32.2	49.2	6.6	–	3.6
Chaumont (Switzerland), rural, year; PM_2.5_ (Hüeglin et al. 2005)	7.7	26	48.2	8.8	–	3.6
Chaumont (Switzerland), rural background, year; PM_2.5_ (Hueglin et al. 2005)	7.7	26	48	9	1	<1
Duisburg (Germany), urban background, autumn; PM_2.5_ (Sillanpää et al. 2006)	14.7	40	39.5	5.6	7.9	1.2
Prague (Czech Republic), urban background, winter; PM_2.5_ (Sillanpää et al. 2006)	29.6	59.7	34.6	2.5	1.1	0.47
Amsterdam (Holland), urban background, winter; PM_2.5_ (Sillanpää et al. 2006)	25.4	28.4	39	2.6	10	0.32
Helsinki (Finland), urban background, spring; PM_2.5_ (Sillanpää et al. 2006)	8.3	54.4	39.3	6.4	5.8	0.35
Barcelona (Spain), urban background, spring; PM_2.5_ (Sillanpää et al. 2006)	20.0	28.6	38.7	4.4	6.0	0.57
Athens (Italy), urban background, summer; PM_2.5_ (Sillanpää et al. 2006)	25.3	41.6	41.4	5.1	1.3	0.34
Mt Cimone (Italy), summer; PM_1_(Marenco et al. 2006)	7.1	46	52	1	<1	<1
Bemantes (Spain), rural background, year; PM_2.5_ (Salvador et al. 2007)	13.5	30	33	11	7	<1
Barcelona (Spain), urban background, year; PM_1_ (Pérez et al. 2008)	19	45	31	5	1	–
Barcelona (Spain), urban background, year; PM_2.5_ (Pérez et al. 2008)	29	34	30	16	2	–
Hyytiälä (Finland), regional background, May; PM_1_ (Saarikoski 2008)	4.4	52	41^b^	–	–	–
Finokalia (Finlandia), coastal, year; PM_1_ (Saarikoski 2008)	12	30	52^b^	–	–	4
Helsinki (Finland), urban, year; PM_1_ (Saarikoski 2008)	21	48	23^b^	–	–	–
Birmingham (UK), urban background, spring/winter; PM_1_ (Yin and Harrison 2008)	12.6^c^	31/49	55/41^d^	5/8^a^	1/2	-
Villar Arzobispo (Spain), rural background, year; PM_2.5_ (Viana et al. 2008a)	18.0	16	28	29	2	<1
Milan (Italy), urban, winter/summer; PM_1_ (Vecchi et al. 2008)	48.8/19.4	*39*/*35*	*41*/*39* ^e^	*1*/*1*	–	<*1*/<*1*
Florence (Italy), urban, winter/summer; PM_1_ (Vecchi et al. 2008)	25.3/11.8	*57*/*27*	*23*/*45* ^e^	<*1*/*2*	–	<*1*/<*1*
Genoa (Italy), urban, winter/summer; PM_1_ (Vecchi et al. 2008)	11.5/17.4	*50*/*33*	*20*/*38* ^e^	*2*/*2*	–	<*1*/<*1*
Montseny (Spain), rural background, year; PM_2.5_ (Pey et al. 2009)	13.6	28	36	9	2	–
Bologna (Italy), rural, summer/winter^f^; PM_1_ (Carbone et al. 2010)	–	48/43^g^	48/56	2/0^a^	2/0	–
Rome (Italy), suburban; summer/winter^f^; PM_1_ (Carbone et al. 2010)	–	62/67^g^	27/28	6/3^a^	5/2	–
Melpitz (Germany), rural background, WEST, summer/winter; PM_1_^h^ (Spindler et al. 2010)	10/11	20/18	<35/55	–	–	–
Melpitz (Germany), rural background, EAST, summer/winter; PM_1_^i^ (Spindler et al. 2010)	17/22	27/25	<32/41	–	–	–
Zabrze (Poland), urban background, summer/winter; PM_2.5_ (Rogula-Kozłowska et al. 2012)	18.4/66.8	54.3/49.6	23.2/15.9	10.6/0.1	1.5/5.8	4.0/8.8

Values in italics are data extracted from a chart

^a^PM rich in Fe + Ca salts

^b^Contribution of water-soluble ions

^c^Average concentration over the whole measuring period

^d^(NH_4_)_2_SO_4_ + (NH_4_NO_3_/NaNO_3_)

^e^(NH_4_)_2_SO_4_ + NH_4_NO_3_

^f^Samples taken during daytime

^g^WSOM (water-soluble organic matter) + WINCM (water-insoluble carbonaceous matter)

^h^Air inflow from western sector

^i^Air inflow from eastern sector

A good method for the rough PM source apportionment is to compare the sum of the analytically determined masses of the groups of the main PM chemical components with the gravimetrically determined PM mass (chemical mass closure). In urban areas, almost the whole mass of PM_2.5_ is the mass of carbon compounds (hundreds of organic compounds), elemental carbon, sulfates, nitrates, ammonia, and geological material (aluminum oxides, silicon, calcium, titanium, iron). Also trace elements, and, in some regions, sodium, and chlorine may be found in PM (Finlayson-Pitts and Pitts 1986; Chow 1995). So, the chemical mass closure is most often checked for the PM components grouped into elemental carbon (EC) and organic matter (OM) or the sum of both (EC + OM), secondary inorganic aerosol (SIA, ambient sulfates, nitrates and ammonium, often corrected for sea salt sulfate content), crustal or mineral matter (CM or MM, main components of Earth crust or both Earth crust and soil), sea salt or marine components (SS or Na_Cl, sea salt components or chlorine and sodium), and trace elements or other elements (TE or OE, trace elements or remaining, not belonging to the other groups, elements or their oxides, Table 1).

In East-Central Europe, the number of sites where PM, especially the finest particles, is adequately studied falls far short of what is needed (e.g., EMEP 2009, 2011; Putaud et al. 2010). In Poland, where the PM_2.5_ content of urban PM is high (Klejnowski et al. 2012), the chemical composition of PM_2.5_ has been so far an object of only few and, in general, short-term studies, confined rather to determinations of the PM elemental composition and concentrations of PM_2.5_ (e.g., Pastuszka et al. 2003, 2010; Zwoździak et al. 2012; Rogula-Kozłowska et al. 2013). However, the shortage of data and the level of air pollution in East-Central Europe, especially in Poland, the Czech Republic, and Ukraine, from where PM migrates to other European regions (Spindler et al. 2010; EMEP 2009, 2011) confirm the need for analyzing PM_2.5_ for more than only its concentrations and elemental composition.

The goal of the presented work was to determine the seasonal variations in the concentrations of PM_2.5_ and PM_2.5_ components, including benzo(*a*)pyrene (BaP), As, Ni, Cd, and Pb, during 1 year in three locations in Poland. The annual concentrations of PM_2.5_ were also determined and its annual mass closure analyzed.

## Materials and methods

### PM_2.5_ sampling and averaging of results

PM_2.5_ was investigated at three sites, very distant from each other: Gdańsk and Diabla Góra in northern Poland, and Katowice in southern Poland (Table 2).

**Table 2:** Sites, equipment, and sampling periods for PM_2.5_ sampling

Measuring sites	Geographic coordinates	Location type	Type and manufacturer of measuring device	Altitude of sampling head (above ground level)	Duration of campaign;averaging time/number of samples
Katowice	50° 15′ 56″ N 18° 58′ 40″ E 274 m a.s.l.	Urban background to agglomerations and/or cities with more than 100000 inhabitants (EC 2008)	PNS-15 (low volume sampler) produced by ATMOSERVICE(Poznań, Poland)	2.5 m	1 January 2010–31 December 2010; 24 h/358
Gdańsk	54° 22′ 49″ N 18° 37′ 13″ E 40 m a.s.l.		DHA-80 (high volume sampler) produced by DIGITEL, (Hegnau, Switzerland)	2.0 m	1 January 2010–31 December 2010; 24 h/356
Diabla Góra	54° 07′ 32″ N 22° 02′ 17″ E 157 m a.s.l.	Rural background (EC 2008)			1 January 2010–31 December 2010; 24 h/365

The station in Katowice is located in a big urban agglomeration in Silesia Province, in the region of the greatest industrial and municipal emissions of PM in Poland (coal-based energy production, densely populated agglomeration; CSO 2011a, b). It is situated within a big urban settlement consisting of low to medium high-rise buildings, about 3.2 km west of the city center. There are also some shops and warehouses south and west of the station and a market place to the west. A highway runs about 500 m south of the station. The station is classified as an urban background station.

The station in Gdańsk is also located in a big urban agglomeration in Pomerania Province, the area of the lowest PM emissions in Poland (CSO 2011a, b). It is affected by road traffic, the Baltic Sea, and industrial and municipal emissions; the effect of the industrial and municipal emissions is much smaller than in Katowice. The station is about 4 km west of the city center. The area is not densely built up within the range of 150 m from the station; the borderline of two settlements consisting of low and medium high-rise buildings is from 350 m east to 500 m north of the station. There are many facilities (university, high schools, hotels, hospitals) nearby and the network of local streets is very dense. About 1 km west of the station, there is a forest area; some industrial areas and sporting facilities are from about 1 to 2 km east. It is an urban background station, like the one in Katowice.

The Diabla Góra sampling site is located in the Borecka Forest, a region of agriculture and forestry, beyond strong anthropogenic effects. There are no air pollution sources in the station neighborhood (no roads, living houses, industry); the nearest object is the forester’s lodge in Diabla Góra, 350 m southwest of the station. It is a rural background station meeting the EMEP background station criteria (Co-operative Programme for Monitoring and Evaluation of the Long-Range Transmission of Air Pollutants in Europe); the air pollution at this site is typical of the whole region (regional background).

During 2010, daily samples of PM_2.5_ were being taken at all the three sites in parallel. PM_2.5_ was collected onto 47- (PNS-15) and 150- (DHA-80) mm-diameter quartz fiber filters (Whatman’s Grade QM-A Circles, cat. no. 1851–047 and cat. no. 1851–150, respectively). Before and after exposing, the filters were conditioned in a weighing room (48 h, relative air humidity 45 ± 5 %, air temperature 20 ± 2 °C) and weighed twice, with a 24-h period between—the former on a Mettler Toledo balance (resolution, 2 μg), the latter on a Sartorius balance (resolution, 100 μg).

Four equal parts were cut out from each, containing a daily PM_2.5_ sample, filter. A monthly PM_2.5_ sample was made by combining together these parts, one from each day in the month of the year. There were four monthly samples for each month, each analyzed for only one compound group: organic (OC) and elemental carbon (EC), water soluble ions (Na^+^, NH_4_^+^, K^+^, Mg^2+^, Ca^2+^, Cl^−^, NO_3_^−^, SO_4_^2−^), metals (As, Ni, Cd, Pb, Ti, Al, Fe), and BaP.

The monthly PM_2.5_ concentrations were computed by dividing the mass of all the daily PM_2.5_ samples from the month by the volume of the air they were taken from.

The PM_2.5_-related substances were determined in the monthly samples. The concentrations for the periods were computed as the averages of the monthly concentrations.

Usually, the ambient concentrations of PM and its components are averaged over a heating or a non-heating period, the year division due to the difference in the air temperatures. In Poland (southern and central-eastern especially), the increased energy demands in a heating period (October–March) yield the increase in the PM emissions from combustion of fossil fuels and biomass. Moreover, the lowest (monthly) air temperatures and the highest concentrations of PM and its precursors occur at the turn of the year (December–January), when the meteorological conditions favor episodes of elevated air pollution or even smog episodes (Pastuszka et al. 2010; Juda-Rezler et al. 2011). Therefore, in 2010, at each location, the concentrations and chemical composition of PM_2.5_ were examined in the heating and non-heating periods (heating: January, February, March, October, November, December; non-heating: April, May, June, July, August, September) and in each season of the year (winter: January, February, December; spring: March, April, May; summer: June, July, August; autumn: September, October, November). The annual concentrations of PM_2.5_ and its chemical components (averages of 12 monthly concentrations) were also computed.

### Chemical analysis

The OC and EC contents were determined using a commercial Behr C50 IRF carbon analyzer (Labor-Technik GmbH, Dusseldorf, Germany). A thermal method (modification of VDI 2465/2) was applied. Heated in nitrogen, a sample exhales OC, oxidized later with copper oxide(II) to carbon dioxide; EC was determined by burning the sample in oxygen. The organic compounds were decomposed successively at the temperatures 200 °C (2 min), 300 °C (2 min 30 s), 450 °C (3 min), and 650 °C (3 min), while elemental carbon was determined at the temperatures 500 °C (2 min), 550 °C (1 min 20 s), 700 °C (2 min), and 850 °C (1 min 20 s).

The CO_2_ content in the gas stream was determined by means of non-dispersive infrared spectrometry (*λ* = 4.26 μm). The gas mixture with certified CO_2_ content was used to calibrate the apparatus. The detection limit for the method was 60.03 μg of CO_2_.

Unlike thermo-optical methods (NIOSH, IMPROVE, or EUSAAR protocols), the used thermal one did not allow for determination of the pyrolytic carbon content (from charring) of total carbon and might yield overestimated EC and underestimated OC contents in PM (Schmid et al. 2001; ten Brink et al. 2004). Indeed, the thermal analysis of the certified reference material (RM 8785 NIST and RM 8786 NIST) gave the standard recoveries equal to 123 % for EC and 71 % for OC of the certified values from the EUSAAR_2 protocol commonly used in Europe.

However, the two methods give close results for TC (total carbon, TC = EC + OC). Besides the reference material, 20 randomly selected PM_2.5_ samples were analyzed for TC with the use of both methods (thermal Behr C50 analyzer; thermo-optical Sunset Laboratory carbon analyzer, Sunset Inc. CA, USA). The differences between the results did not exceed 15 %.

To improve the efficiency of solvent penetration into a sample by reducing the solvent surface tension, the surface of the filters was moistened with 0.1 cm^3^ of ethanol (96 %, pure) before the extraction. The water extracts of PM_2.5_ were made by ultrasonizing the substrates containing samples (a monthly sample) in 50 cm^3^ of deionized water for 60 min at the temperature 15 °C, then shaking for about 12 h (18 °C, 60 r/min). The ion content of extracts was determined using a Metrohm ion chromatograph (Metrohm AG, Herisau, Switzerland). The method was validated against the CRM Fluka products nos. 89316 and 89886; the standard recovery was 92–109 % of the certified value and the detection limits were 0.02 mg/l for NH_4_^+^; 0.05 mg/l for Cl^−^, SO_4_^2−^, and K^+^; 0.07 mg/l for NO_3_^−^ and Na^+^; and 0.12 mg/l for Ca^2+^ and Mg^2+^.

To analyze for metals, a monthly sample was put into a Teflon container and mineralized gradually by successively adding concentrated acids: 12 mL of HNO_3_, 5 mL of HF and 2 mL of HClO_4_, 2 mL of HClO_4_, about 5 mL of H_2_O, and 2 mL of HCl. Cd, As, and Ni were determined in mineralized PM_2.5_ using electrothermal atomic absorption spectrometry according to PN-EN 14902 2006. An Analyst 600 (PerkinElmer, Waltham, MA, USA) with a graphite oven was used. The ammonium (NH_4_H_2_PO_4_) and magnesium (Mg(NO_3_)_2_) matrix modifiers for Cd and Pb, and the mixed palladium–magnesium(Pd(NO_3_)_2_-Mg(NO_3_)_2_) modifier for As and Ni were used. Pb was determined using flame atomic absorption spectroscopy. The remaining metals, Fe, Al, and Ti, were determined with the use of inductively coupled plasma–atomic emission spectroscopy (ICP-AES) on a Varian ICP Liberty 220 spectrometer (Agilent Technologies, Palo Alto, USA). The limits of detection for the method, found by analyzing blanks (clean filter substrates) according to PN-EN 14902, were 1.5 ng/m^3^ for Pb, 0.2 ng/m^3^ for Cd, 0.4 ng/m^3^ for Ni, 0.1 ng/m^3^ for As, 3.15 ng/m^3^ for Ti, 82.6 ng/m^3^ for Al, and 3.24 ng/m^3^ for Fe (average flow rate 55 m^3^/24 h). The recoveries of the NIST SRM 1648a were from 91 % (Ni, Al, Fe) to 101 % (As).

BaP was extracted from the PM_2.5_ samples in dichloromethane (CH_2_Cl_2_) with addition of diatomaceous earth on a Dionex ASE350 extractor (Thermo Fisher Scientific, Sunnyvale, CA, USA). The interfering compounds were removed from the extract using solid phase extraction (Florisil packed columns, temperature and pressure were 100 °C and 10 MPa; total time of heating, extraction, and washing was 0.5 h). The extract was analyzed using high-performance liquid chromatography (HPLC) on an Agilent 1200 chromatograph (Agilent Technologies, Palo Alto, USA) equipped with a diode array and fluorescence detectors, an auto-sampler, and the Chemistation computer system for Windows.

The HPLC set was calibrated using standard solutions prepared by diluting a standard mixture of 16 PAH (including BaP) with dichloromethane (CH_2_Cl_2_) or acetonitrile (C_2_H_3_N). Ultra Scientific PAH Mix PM-611 (100 μg/ml of each PAH in dichloromethane) and PAH Mix 47940 (10 μg/ml of each PAH in acetonitrile) were used. The calibration curve was linear (correlation coefficient was 0.999). The BaP concentration in the extract was determined using an external standard by comparing the peak surfaces (BaP excitation and emission wavelengths were 290 and 450 nm). The BaP detection limit, determined statistically by measuring blanks (clean filter substrates) according to PN-EN 15549, was 0.02 ng/m^3^ (average flow rate 55 m^3^/24 h). The NIST SRM 1649b standard was analyzed to verify the performance of the method. The recovery of the standard was 92 %.

### Chemical mass closure

For the mass reconstruction (chemical mass closure), the PM_2.5_ components were categorized into OM, EC, SIA, CM, Na_Cl, toxic metals (TM), and the rest—unidentified matter (UM).

The masses [EC] and [OC] of EC and OC were computed as [EC] = [EC]_A_/1.23 and [OC] = [OC]_A_/0.71 (according to the recoveries from the standard), where [EC]_A_ and [OC]_A_ are the analytically determined masses of EC and OC. The mass [OM] of OM was assumed to be 1.4 [OC] (Turpin and Lim 2001).

SIA included PM_2.5_-bound SO_4_^2−^, NO_3_^−^, and NH_4_^+^, so [SIA] = [SO_4_^2−^]_A_ + [NO_3_^−^]_A_ + [NH_4_^+^]_A_.

The Na_Cl group included Na^+^ and Cl^−^, therefore [Na_Cl] = [Na^+^]_A_ + [Cl^−^]_A_.

CM included CO_3_^2−^, SiO_2_, Al_2_O_3_, Mg^2+^, Ca^2+^, K_2_O, FeO, Fe_2_O_3_, and TiO_2_; [CM] = [CO_3_^2−^] + [SiO_2_] + [Al_2_O_3_] + [Mg^2+^]_A_ + [Ca^2+^]_A_ + [K_2_O] + [FeO] + [Fe_2_O_3_] + [TiO_2_], where [FeO] and [Fe_2_O_3_] were calculated stoichiometrically from [Fe]_A_ assuming a uniform Fe mass distribution between FeO and Fe_2_O_3_, [SiO_2_] = 2[Al_2_O_3_], and [CO_3_^2−^] = 1.5[Ca^2+^]_A_ + 2.5[Mg^2+^]_A_ (Marcazzan et al. 2001); [Al_2_O_3_], [K_2_O], and [TiO_2_] were calculated stoichiometrically from the analytically determined [Al]_A_, [K^+^]_A_, and [Ti]_A_; [Ca^2+^]_A_ and [Mg^2+^]_A_ were determined analytically.

TM included As_2_O_3_, NiO, CdO, PbO, PbO_2_, and [TM] = [As_2_O_3_] + [NiO] + [CdO] + [PbO] + [PbO_2_], where [PbO] and [PbO_2_] were calculated stoichiometrically assuming uniform Pb mass distribution between PbO and PbO_2_; [As_2_O_3_], [NiO], and [CdO] were calculated stoichiometrically based on the analytically determined [As]_A_, [Ni]_A_, and [Cd]_A_.

UM was PM_2.5_-OM-EC-SIA-Na_Cl-CM-TM, and [UM] = [PM_2.5_]-[OM]-[EC]-[SIA]-[Na_Cl]-[CM]-[TM], where [PM_2.5_] is determined gravimetrically.

The mass closure for PM_2.5_ was examined at each location separately for each season of the year, for the heating and the non-heating periods, and for the whole year 2010.

## Results and discussion

### Concentrations of PM_2.5_

**Fig. 1:**
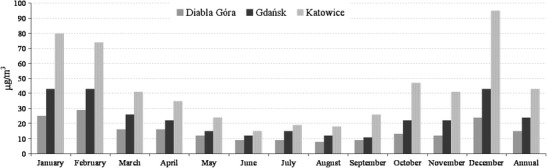
Monthly and annual PM_2.5_ concentrations in three locations in Poland in 2010

In Diabla Góra, the annual concentration of PM_2.5_ was 15 μg/m^3^ (Fig. 1). It was relatively high—the WHO recommends keeping it under 10 μg/m^3^ (WHO 2005), while the EU standard is 25 μg/m^3^ (EC 2008). It was also high compared to the annual concentrations at other EMEP stations (EMEP 2009, 2011). The heating period average PM_2.5_ concentration was higher than the non-heating period one (Table 2). In the non-heating period, the monthly PM_2.5_ concentrations were lower than 16 μg/m^3^ and varied only slightly (in the summer they were almost uniform, Fig. 1). In the winter, they exceeded 24 μg/m^3^; the winter average PM_2.5_ concentration was the highest seasonal PM_2.5_ concentration. The average spring concentration was also high; the autumn and summer ones, not exceeding 11 μg/m^3^, were close to the non-heating period average.

In Gdańsk, the annual PM_2.5_ concentration (24 μg/m^3^) was higher than at the majority of other urban background sites in Europe (EEA 2011, Table 1). As in Diabla Góra, its magnitude was mainly due to the winter, when the monthly concentrations reached their maximum, and in January, February, and December, they exceeded 40 μg/m^3^ (Fig. 1). The summer concentrations were significantly lower; in general, the monthly concentrations were lower in the non-heating than in the heating period. The winter average PM_2.5_ concentration was almost two times the non-heating period average and three times the summer one (Table 3).

**Table 3:** Average concentrations (in microgram per cubic meter) of PM_2.5_ and of PM_2.5_ components in the seasons of a year and in the heating and non-heating periods of 2010 in Katowice, Gdańsk, and Diabla Góra

	PM_2.5_	EC^a^	OC^a^	SO_4_^2−^	NO_3_^−^	Cl^−^	NH_4_^+^	Na^+^	K^+^	Ca^2+^	Mg^2+^	As, Ni, Cd, Pb, Ti, Al, Fe
Katowice												
Spring^b^	33.02	9.83	4.03	2.81	2.44	1.23	2.82	2.78	0.21	0.68	0.13	0.33
Summer^b^	17.29	3.37	2.81	4.41	0.74	0.79	0.72	0.48	0.12	0.24	0.09	0.36
Autumn^b^	38.26	2.49	3.33	4.21	2.46	1.54	1.96	0.44	0.29	0.30	0.06	0.44
Winter^b^	82.97	20.38	12.29	6.89	4.74	3.32	4.22	1.06	0.33	0.21	0.08	0.76
Non-heating^c^	22.73	4.47	3.32	3.78	1.13	0.81	1.59	1.46	0.20	0.45	0.12	0.37
Heating^c^	63.04	13.57	7.91	5.38	4.06	2.63	3.27	0.92	0.27	0.27	0.06	0.58
Gdańsk												
Spring	21.05	2.6	0.21	1.38	2.10	0.56	1.13	1.21	0.09	0.20	0.06	0.38
Summer	13.09	0.87	1.31	2.01	0.51	0.21	0.44	0.48	0.04	0.20	0.04	0.30
Autumn	18.29	1.74	2.18	1.87	1.89	0.47	0.83	1.78	0.14	0.21	0.06	0.16
Winter	42.75	6.1	6.68	4.30	5.64	1.22	3.18	0.85	0.34	0.14	0.09	0.20
Non-heating	14.57	1.23	0.97	1.70	0.79	0.21	0.65	0.65	0.07	0.16	0.05	0.29
Heating	33.02	4.43	4.22	3.08	4.28	1.02	2.13	1.51	0.23	0.21	0.08	0.23
Diabla Góra												
Spring	15.02	2.07	0.57	1.53	1.88	0.14	1.13	1.55	0.12	0.17	0.05	0.11
Summer	8.74	0.44	0.95	2.12	0.30	0.19	0.36	0.75	0.06	0.17	0.04	0.16
Autumn	11.24	0.93	1.39	2.01	1.46	0.20	0.67	1.05	0.10	0.13	0.04	0.10
Winter	25.93	5.19	3.63	4.85	4.77	0.22	2.31	1.81	0.19	0.10	0.03	0.08
Non-heating	10.58	0.99	0.86	1.85	0.65	0.17	0.63	1.19	0.10	0.16	0.04	0.14
Heating	19.88	3.32	2.41	3.40	3.56	0.21	1.60	1.40	0.14	0.13	0.04	0.09

^a^Carbon concentrations (OC and EC) measured thermally (Behr carbon analyzer) may be compared with those measured using thermal-optical method only as total carbon (TC = OC + EC)

^b^Spring: March, April, May; summer: June, July, August; autumn: September, October, November, winter: January, February, December

^c^Non-heating: April, May, June, July, August, September; heating: January, February, March, October, November, December

In Katowice, the annual PM_2.5_ concentration was 43 μg/m^3^ (1.7 times the EU standard), almost two times the one in Gdańsk and three times the one in Diabla Góra. It did not differ much from the concentrations at this site in the previous years (Klejnowski et al. 2009). In the heating period, all the monthly concentrations were greater than 40 μg/m^3^, the December one was maximum.

At each measuring point, the average winter PM_2.5_ concentration was 130 % of the average heating period PM_2.5_ concentration. Except for Katowice, the average summer PM_2.5_ concentrations were close to the average non-heating period ones. In Diabla Góra and Gdańsk, the average concentrations in the non-heating period were two times lower than in the heating period; in Katowice, it was three times. In Diabla Góra and Gdańsk, the summer averages were three times and in Katowice five times lower than the winter averages. These differences reflect the differences between the short and the long periods in the conditions affecting the PM_2.5_ concentrations. In the context of the PM_2.5_ air pollution, they justify considering both the long (heating and non-heating) and the short (spring, summer, autumn, winter) periods of a year, especially when the averaged results are used to assess the health hazard from PM_2.5_.

The great differences in the PM_2.5_ concentrations between winter and summer, or between heating and non-heating periods, at all three sites are an effect of several factors. In Poland, the energy production is based on combustion of hard and brown coal. The flue gases from power plants, which contain precursors of PM_2.5_ (SO_*x*_, NO_*x*_, organic compounds), affect the PM_2.5_ concentrations during a whole year country-wide (more in a heating period), but the main factors affecting the PM_2.5_ concentrations in great cities (Katowice, Gdańsk) are the municipal sources and the atmospheric conditions, unfavorable for pollutant dispersion (Pastuszka et al. 2010; Juda-Rezler et al. 2011). In Diabla Góra, where no local sources of PM_2.5_ exist, the seasonal variations of the PM_2.5_ concentrations are due to the quality of incoming air. The fine particles might have come to Diabla Góra from everywhere, even from very distant regions.

Poland borders on the countries where PM_2.5_ concentrations are also high, and where the emissions from combustion of fossil fuels occur is also a problem. The air from these countries can bring the pollutants to the investigated regions. For the purposes of the present work, to trace the air pollutant transport, 48 h backward trajectories, started at the measuring sites and showing the movements of atmospheric air, were computed.

**Fig. 2:**
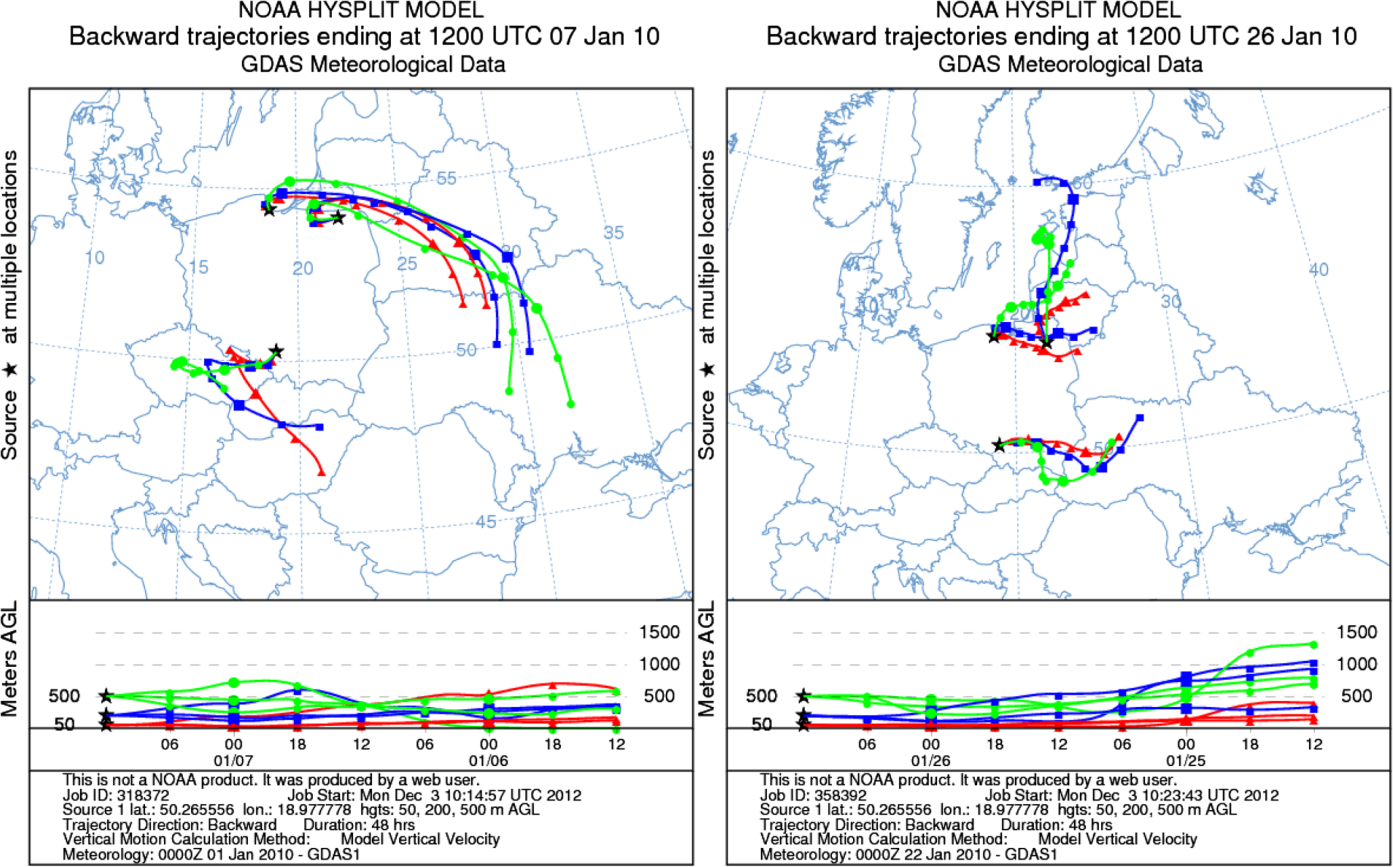
Forty-eight-hour back-trajectories calculated using the HYSPLIT4 model for Katowice, Gdańsk, and Diabla Góra at an altitude of 50 (*red*, *triangles*), 200 (*blue*, *squares*), and 500 (*green*, *circles*) m a.g.l., ending at 12 UTC on 7 and 26 January 2010

The 48 h back-trajectories were computed for several days in January–February 2010 using the HYSPLIT_4 model (Draxler and Rolph 2012; Rolph 2012). They started at the sampling points, 50, 200, and 500 m above the ground level, at 12:00 UTC. Figure 2 presents the back-trajectories computed for 7 and 26 January. They were selected because the daily PM_2.5_ concentrations on these days were, respectively, 199 and 152 μg/m^3^ in Katowice, 62 and 93 μg/m^3^ in Gdańsk, and 40 μg/m^3^ on both days in Diabla Góra.

The low pressure system, centered south of Vilnius, influenced the weather in Poland on 7 January 2010. It was the period of the inflow of cold air from the east into northern Poland and warmer, slightly moister, air from the southwest into southern Poland. Except for the last 6 h, when air came from Gdańsk Bay, the air over Gdańsk and Diabla Góra came from the southeast (middle Ukraine). Light near-surface winds, weak temperature inversion, and mixing layer heights from 250 to 700 m were observed. At the same time, air masses from above the Hungarian Plain moved into the Czech territory, and then north-eastwardly over Katowice. Weak near-surface wind and mixing layer heights, similar to the northern Poland ones, were noted. The effect of remote sources from the neighboring countries on the PM_2.5_ concentrations on 7 January 2010 at the three sites cannot be excluded.

The back-trajectory for 26 January indicated air inflow from northern or north-eastern Europe. To Gdańsk, the air masses came from over the Baltic Sea; to Diabla Góra, they travelled through the coastal regions of Kaliningrad Oblast and of the Baltic Countries (Estonia, Latvia, Lithuania). Aerological soundings confirmed strong temperature inversion on 26 January. The mixing layer height varied between 100 and 400 m in Katowice and did not exceed 400 m in Gdańsk and Diabla Góra. The most stable atmospheric class was noted at all the three locations (Pasquill 1961). Despite the air inflow from other regions, and possible effect of remote sources, the shallow mixing layers, inversion, and isothermy suggest rather attributing all the ambient particulate matter to local and/or regional sources on 26 January 2010 at all three sites.

### Chemical composition of PM_2.5_

**Fig. 3:**
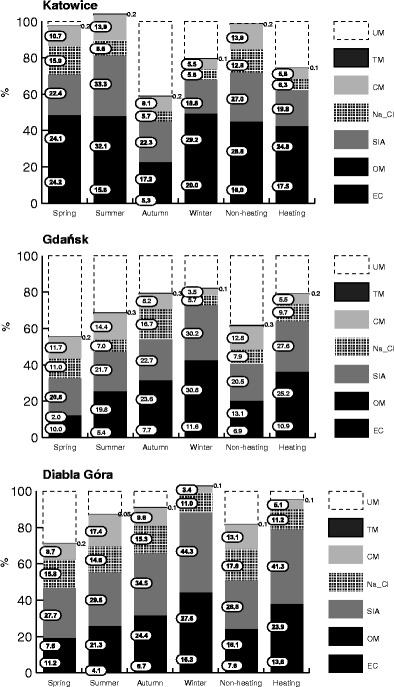
The seasonal percentages (contributions) of the main groups of the chemical components in PM_2.5_ (*EC*, elemental carbon; *OM*, organic matter; *SIA*, secondary inorganic aerosol; *Na_Cl*; *CM*, crustal matter; *TM*, toxic metals) and unidentified matter (UM) in PM_2.5_ at three sampling sites in Poland

The mass shares of the identified components in PM_2.5_ were from 58.8 % (autumn) to 103.9 % (winter) in Katowice, from 55.4 % (spring) to 81.9 % (winter) in Gdańsk, and from 70.6 % (spring) to 102.7 % (winter) in Diabla Góra (Fig. 3). The unidentified matter contributed more to the PM_2.5_ mass in the non-heating than in the heating period in Gdańsk and Diabla Góra, in Katowice—conversely.

UM in PM_2.5_ may include compounds that were not covered by the analyses (many trace elements, carbonaceous, and/or organic anions) or evaporated during storage and transportation of samples (some organic compounds and nitrates, especially in hot and dry periods). It may also contain water. In Europe, the water content of PM_2.5_ at the 50 % relative air humidity may vary from 20 to 30 %, depending on a place (Tsyro 2005). The unknown amount of water in PM_2.5_-related hygroscopic compounds (ammonium nitrate, chlorides, compounds of sodium, Table 4) may account for a considerable part of UM in the periods of high precipitation and air humidity—as in the spring in Gdańsk and Diabla Góra, and in the autumn in Katowice. In turn, the adsorption of SO_2_ onto particles rich in ammonia or adsorption of nitric acid (HNO_3_) onto mineral and salt particles on a filter (Pathak et al. 2009) may have caused an overestimation of the identified part of PM_2.5_ (Katowice—summer, Diabla Góra—winter).

**Table 4:** The equivalent ion (Σ_cations_/Σ_anions_,), neutralization ratio (NR), molar ratios (SO_4_^2−^/NH_4_^+^, SO_4_^2−^/Na^+^, Cl^−^/Na^+^, Mg^2+^/Na^+^, K^+^/Na^+^), and estimated concentrations of (NH_4_)_2_SO_4_ and NH_4_NO_3_ in the seasons of the year and the heating and non-heating periods in Katowice, Gdańsk, and Diabla Góra

	Σ_cations_/Σ_anions_^a^	NR^b^	SO_4_^2−^/NH_4_^+c^	[(NH_4_)_2_SO_4_]^d^ [μg/m^3^]	[ex-NH_4_^+^]^e^ [μg/m^3^]	[NH_4_NO_3_]^f^ [μg/m^3^]	SO_4_^2−^/Na^+c^	Cl^−^/Na^+c^	Mg^2+^/Na^+c^	K^+^/Na^+c^
Katowice										
Spring^g^	2.46	1.60	0.37	3.88	1.77	7.80	0.48	0.29	0.04	0.04
Summer^g^	0.79	0.39	2.30	2.64	–	–	4.40	1.08	0.18	0.15
Autumn^g^	0.91	0.85	0.81	5.81	0.39	1.72	4.58	2.30	0.13	0.39
Winter^g^	0.97	1.07	0.61	9.51	1.65	7.27	3.11	2.06	0.07	0.18
Non-heating^h^	1.58	0.91	0.89	5.22	0.18	0.80	1.24	0.36	0.08	0.08
Heating^h^	0.98	1.02	0.62	7.42	1.27	5.57	2.80	1.88	0.06	0.17
Gdańsk										
Spring	1.69	1.00	0.46	1.90	0.62	2.71	0.55	0.30	0.05	0.04
Summer	1.06	0.49	1.71	1.61	–	–	2.01	0.29	0.08	0.05
Autumn	1.72	0.66	0.84	2.58	0.13	0.59	0.50	0.17	0.03	0.05
Winter	1.10	0.98	0.51	5.93	1.58	6.94	2.42	0.94	0.10	0.24
Non-heating	1.45	0.75	0.98	2.35	0.02	0.07	1.25	0.21	0.07	0.06
Heating	1.28	0.89	0.54	4.25	0.98	4.32	0.98	0.44	0.05	0.09
Diabla Góra										
Spring	2.20	1.01	0.51	2.11	0.56	2.46	0.47	0.06	0.03	0.05
Summer	1.21	0.41	2.21	1.32	–	–	1.35	0.17	0.05	0.05
Autumn	1.34	0.57	1.13	2.46	–	–	0.92	0.13	0.04	0.06
Winter	1.19	0.72	0.79	6.69	0.50	2.21	1.28	0.08	0.02	0.06
Non-heating	1.87	0.71	1.10	2.31	–	–	0.74	0.09	0.03	0.05
Heating	1.22	0.69	0.80	4.69	0.33	1.47	1.16	0.10	0.03	0.06

^a^The equivalent ion balance, expressed as the ratio of total cation equivalent to total anion equivalent

^b^NR = NH_4_^+^/(SO_4_^2−^ + NO_3_^−^) where NH_4_^+^, SO_4_^2−^, and NO_3_^−^ are in normal equivalents per cubic meter

^c^Molar ratios

^d^[(NH_4_)_2_SO_4_] = 1.38[SO_4_^2−^]_A_ if SO_4_^2−^/NH_4_^+^ <1 and [(NH_4_)_2_SO_4_] = 3.67 [NH_4_^+^]_A_ if SO_4_^2−^/NH_4_^+^ >1

^e^[ex-NH_4_^+^] = [NH_4_^+^]-0.27[(NH_4_)_2_SO_4_]

^f^[NH_4_NO_3_] = 4.44[ex-NH_4_^+^]

^g^Spring: March, April, May; summer: June, July, August; autumn: September, October, November, winter: January, February, December

^h^Non-heating: April, May, June, July, August, September; heating: January, February, March, October, November, December

**Fig. 4:**
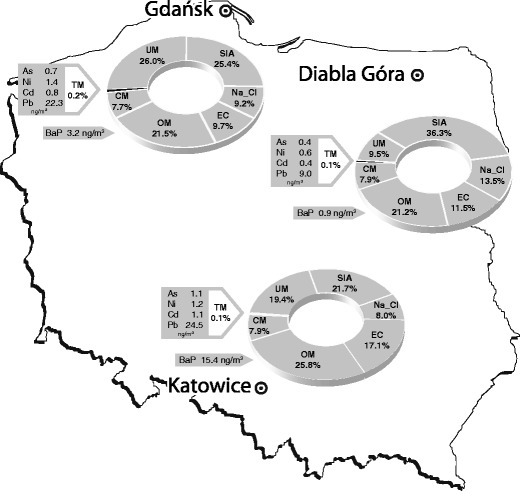
Annual shares of the main groups of the chemical components in PM_2.5_ and annual concentrations of PM_2.5_-related BaP, As, Ni, Cd, and Pb in three locations in Poland (*EC*, elemental carbon; *OM*, organic matter, *SIA*, secondary inorganic aerosol; *Na_Cl*; *CM*, crustal matter, *TM*, toxic metals; *UM*, unidentified matter)

In Katowice, the mass contribution of carbon compounds (OM + EC) to PM_2.5_ was between 42 and 49 % in all the seasons except for the autumn. The similar high OM + EC in PM_2.5_ was noted in Zabrze, 15 km west of Katowice, in 2009 (Fig. 3 and Table 1). In Gdańsk, the OM + EC contributions to PM_2.5_ were from 12 % (spring) to 42 % (winter), in Diabla Góra—from 19 % (spring) to 44 % (winter, Fig. 3). The annual mass contributions of OM + EC to PM_2.5_ were 43, 31, and 33 % in Katowice, Gdańsk, and Diabla Góra, respectively (Fig. 4).

At the European urban background sites, OM + EC contributes to the PM_2.5_ mass from about 29 % (Barcelona, Spain) to 60 % (Prague, winter). The contributions are significantly smaller at the rural background sites—from 16 % (Villar Arzobispo in Spain) to about 30 % (Bemantes and Montseny in Spain, Melpitz in Germany, Table 1).

In Poland, as elsewhere in Europe, the mass of OM + EC in PM_2.5_ is much lower within near-sea urban areas than at urban background sites distant from a coast. In Gdańsk, a coastal urban area, the contribution is even lower than in rural Diabla Góra. The contributions tend to be lower in spring and higher in winter both in Poland and other European regions. In Katowice, the autumn OM + EC mass contribution to PM_2.5_ was distinctly lower than the other, not differing much from each other, seasonal ones (Fig. 3).

Nevertheless, at all three sites, the heating period averages of the concentrations of PM_2.5_-related both OC and EC were higher than the non-heating season ones, and among the seasonal ones, the winter averages were highest (Table 3). The heating period average of the ambient concentration of EC was about three times greater than the non-heating period one; the proportions of the heating to the non-heating period averages of OC were from 2.4 in Katowice to 4.3 in Gdańsk. The winter averages of the OC and EC concentrations were higher than the heating period ones by 37–58 %.

EC is mainly emitted as primary soot from combustion; therefore, its higher ambient concentrations in winter or in a whole period of intensified combustion of fossil fuels all over Poland are obvious. However, in Katowice, where domestic combustion and intense road traffic maintain high EC concentrations during the whole year, the ambient spring, summer, and autumn EC concentrations were also very high (summer average of the EC concentration in Katowice was almost four and eight times greater than in Gdańsk and Diabla Góra, respectively; Table 3). EC, released mainly as very fine particles (mass median diameter about 2 μm, Japar et al. 1986), has a very long residence time in the air and may travel long distances. This explains the higher ambient EC concentrations in the heating than in the non-heating period in Diabla Góra where there are no local sources of PM_2.5_-related EC. The fine particles of soot from power stations (high stacks) might have come to Diabla Góra from other regions, even from abroad (Fig. 2, 7 January).

In a heating period (in winter especially), when the naturally low insolation is additionally suppressed by high ambient concentrations of soot, the greater part of ambient organic matter probably consists of primary compounds from combustion and biological material. In the warmer periods, the OM concentrations at sampling sites may be attributed to the enhanced formation of secondary organic aerosol (Seinfeld and Pandis 2006). However, the indirect confirmation of this fact (e.g., by using the method from Castro et al. 1999) requires the ratio OC/EC determined at each site. With the method used (thermal carbon analyzer without optical correction for separation of charred organic carbon), it is impossible.

The water-soluble ions (especially SO_4_^2−^, NO_3_^−^, NH_4_^+^; in Katowice also Na^+^ and Cl^−^) followed EC and OM in decreasing order of the mass shares in PM_2.5_ at all three sites (Table 3, Figs. 3 and 4). In urban areas, the mass of sulfates (SO_4_^2−^), nitrates (NO_3_^−^), and ammonium (NH_4_^+^) may reach 85 % of all the water-extractable ions in PM_2.5_ and 15–55 % of the total mass of PM_2.5_ (Table 1).

The annual mass contributions of SIA (SO_4_^2−^, NO_3_^−^, NH_4_^+^) to PM_2.5_ were 22, 25, and 36 % in Katowice, Gdańsk, and Diabla Góra, respectively. All the short and long period averages of the mass SIA contributions to PM_2.5_ were between 19 % (Katowice, winter) and 44 % (Diabla Góra, winter, Fig. 3).

In Gdańsk and Diabla Góra, the concentrations of SIA and its mass contributions to PM_2.5_ were lower in the non-heating than in the heating period (elevated winter emissions of the precursors all over Europe, higher ambient concentrations of the SIA components at each location in the heating period, especially in the winter). In Katowice, the SIA contribution to PM_2.5_ was high in the summer—almost 33 %. In the heating period, the share of SIA in PM_2.5_ was low, not greater than 20 %. However, while the mass contribution of SIA to PM_2.5_ in Katowice was lower than at the two other sites, the season- and the period-averaged ambient concentrations of the components of SIA (SO_4_^2−^, NO_3_^−^, and NH_4_^+^) were higher (Table 3). It is due to the PM_2.5_ precursors, SO_*x*_ and NO_*x*_, coming from the (whole year) combustion of hard and brown coal for energy production all over Poland, exceptionally intense in southern Poland.

In summer (higher temperatures and lower air humidity), reaction of ammonia with sulfuric acid or ammonium bisulfate is favored over reaction with nitric acid (Seinfeld and Pandis 2006). Proportions of the ionic equivalent concentrations of SO_4_^2−^ and NH_4_^+^ are greater than 1 at each site (SO_4_^2−^/NH_4_^+^ >1, Table 4); therefore, it is probable that ammonium nitrate (NH_4_NO_3_) did not occur in SIA at any site in the summer. Hence, in the summer, the atmospheric aerosol at all three sites might have been acidic. The PM_2.5_ acidity within the investigated regions is also confirmed by low neutralization ratios (Table 4) at all sites in the summer. Some amounts of ammonium nitrate might have evaporated, especially from the samples taken in the summer, but the artifacts related to the semi-volatility of ammonium nitrate can be of importance only for the samples very rich in ammonium (Pathak et al. 2009). In general, the acidity of PM_2.5_ cannot be excluded at any site not only in the summer but also in the autumn and in the winter. It is a common assumption that if NH_4_^+^/SO_4_^2−^ ≥1.5 (molar ratio) then sulfuric acid is totally neutralized (Seinfeld and Pandis 2006), but some authors report the PM_2.5_ acidity even when NH_4_^+^/SO_4_^2−^ ≥2 (in fact, when SO_4_^2−^/NH_4_^+^ ≤0.5; Pathak et al. 2009; Huang et al. 2011). The low proportions of the concentrations of total cations to total anions (equivalent ion balance, Σ_cations_/Σ_anions_, Table 4) at all sites and seasons, except for the spring, also confirm the acidity of PM_2.5_.

The highest Σ_cations_/Σ_anions_ occurred at all sites in the spring (spring and autumn values were almost equal in Gdańsk), when the average ambient concentrations of NH_4_^+^ were high, and the concentrations of SO_4_^2−^ and NO_3_^−^ were much lower than in the winter and autumn (Table 3). In Katowice, Gdańsk, and Diabla Góra, the contributions to SIA of ammonium sulfate ((NH_4_)_2_SO_4_) were 48, 41, and 46 %, and of ammonium nitrate (NH_4_NO_3_)—44, 35, and 33 %, respectively (Tables 3 and 4). Therefore, in contrast to the summer, the atmospheric aerosol was alkaline in the spring, and the contribution of ammonium nitrate to SIA was high at all three sites (close to that of ammonium sulfate).

In the winter, SIA consisted of (NH_4_)_2_SO_4_ and NH_4_NO_3_ at all three sites because of the efficient neutralization of nitrates and sulfates (concentrations of NH_4_^+^ were from six to eight higher than in the summer). Their average contributions to SIA were in the proportion 2:1 both in Katowice and in Diabla Góra; in Gdańsk, the average contribution of NH_4_NO_3_ to SIA was 75 %.

In the winter, the stable atmospheric conditions, stagnation of air masses, and shallow mixing layer favored the accumulation of pollutants and condensation of semi-volatile species, causing high PM episodes, mainly accounted for by carbonaceous matter and ammonium nitrate (typical of urbanized areas of continental Europe, Table 1). Furthermore, the differences in the ammonium sulfate concentrations between stations are much smaller than in those of ammonium nitrate, indicating that the latter is more influenced by local emissions in cities (probably traffic), whereas background aerosol is enriched with ammonium sulfate.

In the autumn, when ambient sulfates and nitrates were high relative to NH_4_^+^ (Table 3), and the conditions were unfavorable for the creation of ammonium nitrate, the shares of ammonium nitrate in SIA in Katowice and Gdańsk were 20 and 6 %, respectively, and it was not found in SIA in Diabla Góra (Table 4).

The total neutralization of sulfuric acid is signalized by the value of NH_4_^+^/SO_4_^2−^ (or SO_4_^2−^/NH_4_^+^); therefore, the simple stoichiometric computations under the assumption that if NH_4_^+^/SO_4_^2−^ ≥1 then all sulfates react with ammonium ions resulted in the overestimation of the autumn ambient concentrations of (NH_4_)_2_SO_4_ in Gdańsk and Katowice and the winter ones in Diabla Góra.

For all three locations, the conclusions about the SIA formation in the non-heating period resemble those about the autumn. The conclusions drawn for the heating season (Table 4) are the same as for the winter in Katowice and Diabla Góra and for the spring in Gdańsk. It is a strong argument in favor of analyzing whole year data sets divided into four subsets matching the seasons of the year, because, for example, in summer the composition or the pH of SIA can entirely differ from the ones in other seasons.

The annual mass contributions of the Na_Cl group to PM_2.5_ were 8, 13.5, and 9.2 % in Katowice, Diabla Góra, and Gdańsk, respectively (Fig. 4). At each site, they were smaller than the contributions of SIA, which, in turn, were smaller than the contributions of OM + EC in Gdańsk and Katowice. Among the seasonal averages of the Na_Cl contributions, the spring and summer averages are greatest in Katowice, and the spring and autumn ones in Gdańsk and Diabla Góra (Fig. 3). In Gdańsk, the high autumn and spring mass contributions of Na_Cl to PM_2.5_ are due to high ambient concentrations of Na^+^ and Cl^−^. It may be the effect of the sea—the spring and autumn winds increase the amounts of dispersed seawater in the air; also SO_4_^2−^/Na^+^ and K^+^/Na^+^ were lowest in Gdańsk in these seasons (Table 4, for seawater they are equal to 0.252 and 0.036, respectively; Seinfeld and Pandis 2006). The remaining molar ratios were entirely different from the ratios for seawater.

The station in Diabla Góra is 140 km from the Baltic Sea. The sea effect on the ambient Na_Cl concentration cannot be excluded (considering the values of SO_4_^2−^/Na^+^ and K^+^/Na^+^, especially in spring, Table 4), but it would be moderated by the western and south-western winds prevailing in this region. The Na_Cl mass contributions to PM_2.5_ in the summer and in the autumn, high in average, are due to the high Na^+^ concentrations. The seasonal variations of the Cl^−^ concentrations were not as high as the variations of the Na^+^ concentrations (Table 3). In Diabla Góra, the ambient concentrations of TC (TC = EC + OC) and K^+^ were high in the spring. Probably, a source of alkaline substances appeared nearby, possibly biomass burning (grass fires).

In spring and early summer, PM_2.5_ from grass fires or biomass combustion in neighboring green areas and allotment gardens might be a source of alkaline substances in Katowice. Indeed, in 2010, the winter Na_Cl concentrations in Zabrze (about 15 km from Katowice) and in Katowice were almost equal (Rogula-Kozłowska et al. 2012, Table 3), while in the summer 2010 the one in Katowice was over eight times that in Zabrze. In Katowice (460 km from the Baltic Sea, south-western winds prevailing), where the concentrations of PM_2.5_ and of its components are due rather to local factors (urban conditions, industry, dense population, and road traffic), and where the distance between the measuring point and the nearest road excludes the influence of street salting, Na_Cl in the winter came from combustion (residential heating or industrial activities; Rogula-Kozłowska et al. 2013).

The variations of the seasonal ambient concentrations of Cl^−^ (concentrations higher in the heating than in the non-heating period, highest in the winter, Table 3), good linear correlation between monthly concentrations of Cl^−^ and EC (*R* ^2^ = 0.61) confirm the conclusion. Besides the low quality coal, also household garbage and wastes burnt in domestic stoves may account for the high concentrations of PM_2.5_-bound Cl^−^ in the winter in Katowice (Chow 1995; Rogula-Kozłowska et al. 2012).

The contributions of CM to PM_2.5_ behaved similarly at all three sites; all the annual ones were about 8 % (Fig. 4). They resemble the contributions in other regions (Table 1). At all the sites, CM contributed to PM_2.5_ more in the non-heating than in the heating period (Fig. 3). Among the seasonal mass contributions, the summer and spring ones were highest in Gdańsk and Katowice; in Diabla Góra—the summer and autumn ones. The contributions were smallest in the winter (not greater than 5.5 %). As it has been mentioned, some part of ambient K^+^ (one of the basic elements in CM) may come from combustion, especially in spring and summer when biomass is burnt. In winter, K, Ca, Mg, Al, and Fe from combustion (also of fossil fuels) may occur in the atmosphere. These elements, and also Ti and Al, are released from transport and some industries all year round (Chow 1995).

The possible anthropogenic effect on the ambient concentrations of K, Ca, Mg, Ti, and Fe can be assessed by computing enrichment factors (EF, Table 5). The enrichment factor EF_*x*_ for the element *x* is defined as:

**Table 5:** Enrichment factors (EF) for PM_2.5_-related Mg, K, Ca, Ti, Fe, As, Cd, Ni, and Pb in three locations in Poland in 2010

		EF_Mg_^a^	EF_K_^a^	EF_Ca_^a^	EF_Ti_	EF_Fe_	EF_As_	EF_Cd_	EF_Ni_	EF_Pb_	Al, ng/m^3^
Katowice	Winter	1	2	1	1	1	312	4,852	28	832	457
	Spring	6	5	15	2	3	468	3,101	72	1,778	120
	Summer	3	2	4	2	3	349	7,592	90	678	154
	Autumn	2	4	4	1	2	389	5,598	43	913	224
	Heating	1	2	2	1	1	361	4,928	34	923	322
	Non-heating	4	3	8	2	3	344	5,915	75	991	155
Gdańsk	Winter	16	30	12	3	10	1,667	51,368	170	6,425	31
	Spring	2	1	2	1	2	87	957	27	516	214
	Summer	1	1	4	1	2	90	1,869	65	176	142
	Autumn	6	6	9	1	3	329	5,394	44	1,129	65
	Heating	5	8	7	1	3	463	11,919	53	1,829	80
	Non-heating	2	1	3	1	2	101	1,719	50	389	146
Diabla Góra	Winter	6	15	8	2	2	906	19,778	67	2,690	34
	Spring	4	4	6	1	1	119	3,293	18	492	78
	Summer	2	1	4	1	1	70	1,186	34	81	128
	Autumn	3	4	5	<1	1	188	1,846	24	316	74
	Heating	4	7	6	1	1	439	7,335	41	1,157	54
	Non-heating	2	3	4	1	1	75	1,984	26	187	103

^a^Computed from concentrations of Mg^2+^, K^+^, and Ca^2+^

1 $$ {\mathrm{EF}}_x=\frac{{\left({C}_x/{C}_{\mathrm{ref}}\right)}_{\mathrm{PM}}}{{\left({C}_x/{C}_{\mathrm{ref}}\right)}_{\mathrm{crust}}} $$

where *C*_*x*_ and *C*_ref_ are the concentrations of the element *x* and the reference element, and (*C*_*x*_/*C*_ref_)_PM_ and (*C*_*x*_/*C*_ref_)_crust_ are the proportions of these concentrations in PM and in the Earth crust, respectively. Al is the reference element, i.e., EF_Al_ = 1. The Al concentrations for all locations and periods are given in the last column of Table 5. The chemical composition of the upper continental crust was taken from Wedepohl (1995).

The closer EF of an element to 1, the weaker the anthropogenic effect on the element ambient concentrations.

The analysis of EF shows that in some seasons of the year (in Katowice in spring; in Diabla Góra in spring, autumn, and winter; in Gdańsk in winter) an anthropogenic source of elements belonging to CM probably appeared. This source affected the concentrations of magnesium, potassium, and calcium most. On the other hand, weak variability of EF and CM content of PM_2.5_ among the sampling sites and seasons of the year mean that this possible (other than soil) anthropogenic source of the crustal elements has no significant effect on the ambient concentrations of the elements at the three sites. It would prove the adequacy of the method (commonly applied in such investigations) assumed for assessing the CM contribution to PM_2.5_.

Compared to the other components, the TM contribution to PM_2.5_ was small (0.05–0.4 %) at all the sampling sites and in all the seasons and periods (Fig. 3). In Gdańsk and Katowice, it was higher in the non-heating than in the heating period, but this was not due to the collective ambient concentrations of the elements from TM (the TM ambient concentrations were two times greater in the heating than in the non-heating period in Katowice and Diabla Góra), but to the lower PM_2.5_ concentrations in the non-heating period.

The average ambient concentrations of As, Cd, and Pb were higher in the heating period at all the sites; they were highest in the winter. The Ni average ambient concentrations were higher in the non-heating period at all the sites; the summer ones were highest (Table 6).

**Table 6:** Ambient concentrations of PM_2.5_-related As, Cd, Ni, and Pb in Katowice, Gdańsk, Diabla Góra, and other European cities

Location	Sampling period		Concentration, ng/m^3^			
			As	Cd	Ni	Pb
Katowice (Poland), urban background^a^	Winter	2010	3.68	2.92	3.12	83.46
	Spring		1.45	0.49	2.08	46.83
	Summer		1.39	1.54	3.34	22.92
	Autumn		2.25	1.65	2.29	44.87
	Heating		3.00	2.09	2.62	65.25
	Non-heating		1.38	1.21	2.80	33.79
	Winter		1.33	2.09	1.26	43.57
	Spring		0.48	0.27	1.40	24.26
Gdańsk (Poland), urban background^a^	Summer		0.33	0.35	2.22	5.49
	Autumn		0.55	0.46	0.68	16.05
	Heating		0.96	1.26	1.02	32.23
	Non-heating		0.38	0.33	1.76	12.45
	Winter		0.79	0.88	0.54	19.95
	Spring		0.24	0.34	0.34	8.46
Diabla Góra (Poland), rural background^a^	Summer		0.23	0.20	1.05	2.27
	Autumn		0.36	0.18	0.43	5.13
	Heating		0.61	0.52	0.53	13.67
	Non-heating		0.20	0.27	0.65	4.23
Zabrze (Poland); urban background^b^	Winter 2009		9.52	1.14	0.36	49.71
Milan (Italy); residential-commercial area^c^	Winter 2002		3	–	9	55
Menen (Belgium); suburban/industrial^d^	Winter 2003		–	–	3.4	54
Athens (Greece); suburban^e^	October–November 2003		6.83	0.70	1.59	5.45
Cartagena (Spain); suburban^f^	January–March 2005		0.3	0.2	3.4	5.7
Barcelona (Spain); urban background^g^	2005–2006		0.6	0.3	3.0	17
Göteborg (Sweden) urban background^h^	February–March 2005		–	–	5.6	6.2
rural site^h^	February 2005		–	–	2.7	5.7

^a^This study

^b^Rogula-Kozłowska et al. (2012)

^c^Vecchi et al. (2004)

^d^Ravindra et al. (2008)

^e^Vasilakos et al. (2007)

^f^Negral et al. (2008)

^g^Pérez et al. (2008)

^h^Boman et al. (2009)

The EF (Table 5) illustrate the anthropogenic effect on the concentrations of PM_2.5_-bound elements.

EF of As, Cd, Pb, and Ni are significantly greater than 1 (EF of Ni is lower than the rest by one order, Table 5) and indicate the anthropogenic origin of these toxic metals in all the periods and at all the sites. The anthropogenic effect on their ambient concentrations is uniform over all the seasons in Katowice and is higher in the heating than in the non-heating period in Gdańsk and Diabla Góra. In Katowice, this (apparent) seasonal independence of the anthropogenic effect was partially due to the seasonal changes in the concentrations of PM_2.5_-bound Al (reference element, Table 5). In Katowice, in a heating period, the ambient concentrations of typical crustal elements (such as Al) may be affected by the emission from coal combustion (municipal sources or power plants; Xu et al. 2003; Yi et al. 2008).

Not surprisingly, the seasonal and periodic concentrations of As, Cd, Ni, and Pb were lower at the rural site in Diabla Góra than in the great cities, Gdańsk and Katowice, but they were comparable with the winter ones at the suburban sites in Spain and Greece, and at the rural site in Sweden (Table 6). The winter concentrations of these metals (especially Pb and Cd) at the two Polish urban sites (and in Zabrze, close to Katowice) were high compared to other European urban sites. Nevertheless, this comparison of probably the highest (because in cold periods) ambient concentrations of As, Cd, Ni, and Pb in several urban and sub-urban European areas with the concentrations in Gdańsk, Katowice and Diabla Góra points out the similarity of the PM_2.5_-bound toxic metal air pollution at the three Polish sites and elsewhere in Europe, although Upper Silesia (Katowice, Zabrze) is the most industrialized region in Poland.

Like those of PM_2.5_, the ambient concentrations of PM_2.5_-related BaP were higher in the heating (very high in the winter) than in the non-heating period in all three locations (Table 7). In Katowice and Gdańsk, they were very high (highest in Katowice, up to almost 36 ng/m^3^ in the winter), but even in rural Diabla Góra the ambient BaP concentrations were higher than in other European cities in the winter (except Zagreb and Prague). At all the three locations, the high annual BaP concentrations (Fig. 4) derived from the very high concentrations in the winter and high concentrations in the spring and autumn. In Katowice, the annual BaP concentration was several times the BaP annual limit (1 ng/m^3^, EC 2004, Fig. 4). Very high BaP concentrations were also observed in the winter 2006/2007 in Zabrze (Table 7) but they were much lower than in Katowice in 2010.

**Table 7:** Ambient concentrations of PM_2.5_-related BaP in Katowice, Gdańsk, Diabla Góra, and other European cities

Location	Sampling period		Concentration BaP, ng/m^3^
Katowice (Poland), urban background^a^	Winter	2010	35.93
	Spring		5.53
	Summer		0.79
	Autumn		11.42
	Heating		24.62
	Non-heating		2.21
Gdańsk (Poland), urban background ^a^	Winter		9.67
	Spring		1.34
	Summer		0.14
	Autumn		1.74
	Heating		6.04
	Non-heating		0.41
Diabla Góra (Poland), rural background^a^	Winter		2.43
	Spring		0.35
	Summer		0.05
	Autumn		0.69
	Heating		1.66
	Non-heating		0.1
Zabrze (Poland), urban background^b^	Winter 2006/2007		6.27
Duisburg (Germany), urban background^c^	September–November 2002		1.05
Prague (Czech), urban background^c^	November 2002–January 2003		3.03
Amsterdam (Netherlands), urban background^c^	January–March 2003		0.33
Zagreb (Croatia)^d^	Winter 2004		3.18
Kozani (Greece); urban area^e^	December 2005–October 2006		0.09
Virolahti (Finland), regional background^f^	February 2006		0.69
Augsburg, (Germany) urban aerosol^g^	February–March 2008		0.83
Kaunas (Lithuania)^h^	Winter 2009		3.2; 6.2

^a^This study

^b^Klejnowski et al. (2010)

^c^Saarnio et al. (2008)

^d^Šišović et al. (2005)

^e^Evagelopoulos et al. (2010)

^f^Makkonen et al. (2010)

^g^Pietrogrande et al. (2011)

^h^Kliucininkas et al. (2011)

Majority of PAH are anthropogenic (in urban areas especially); they come mainly from fossil fuel and biomass combustion, waste incineration, industry, and traffic (e.g., Khalili et al. 1995). So, in Polish cities, especially in southern Poland where municipal and industrial emissions are significant, the concentrations of PM_2.5_ and PM_2.5_-related BaP are very high (e.g., Katowice). The PM_2.5_ and PM_2.5_-related BaP concentrations in the non-heating period, higher in Katowice than in Gdańsk and Diabla Góra or other European regions, confirm the strong effects of industry (cokery, power production, steel industry) in southern Poland.

The highest ambient BaP concentrations, reaching tens of nanogram per cubic meter, occur in densely populated urban regions of Poland (Katowice, Zabrze, Gdańsk) and in the neighboring countries (the Czech Republic, Lithuania, Croatia) where, as in Poland, the anthropogenic (municipal in winter) emissions prevail. It means a great health hazard from ambient BaP and other PAH to the inhabitants, and the greatest hazard in Europe occurs probably in southern Poland (Table 7).

## Summary and conclusions

At each of the three sampling points, the PM_2.5_ concentrations were high compared to other similar European sites beyond Poland. The annual average PM_2.5_ concentration in Katowice (southern Poland, urban background) in 2010 was almost two times greater than the target permissible value established for the EU countries. It was also twice the annual PM_2.5_ concentration in Gdańsk, and thrice the one in Diabla Góra (northern Poland, urban and regional background, respectively). The high annual averages were due to very high monthly averages in the heating period, especially in the winter.

At each site, PM_2.5_ consisted mainly of compounds of carbon (organic matter, OM, and elemental carbon, EC), SIA, the Na_Cl group, and CM—in the decreasing order of their mass contributions to PM_2.5_.

Carbonaceous matter (OM + EC) contributed most to PM_2.5_ in the winter—49.2, 42.4, and 43.9 % in Katowice, Gdańsk, and Diabla Góra; the annual average mass contributions were 43, 31, and 33 %, respectively.

The annual average mass contributions of SIA (including SO_4_^2−^, NO_3_^−^, and NH_4_^+^) to PM_2.5_ were 22, 25, and 36 % in Katowice, Gdańsk, and Diabla Góra. Its highest seasonal mass contribution to PM_2.5_ was 44 % (Diabla Góra, winter), the lowest—19 % (Katowice, winter). At each location, SIA comprised both (NH_4_)_2_SO_4_ and NH_4_NO_3_ in the winter; NH_4_NO_3_ was not found in SIA at any site in the summer.

The Na_Cl group was 8, 13.5, and 9.2 % of the PM_2.5_ mass in Katowice, Diabla Góra, and Gdańsk, respectively. Among its seasonal average contributions, the spring and the summer ones were highest in Katowice and the spring and autumn ones in Gdańsk and Diabla Góra.

The contribution of CM to the PM_2.5_ mass did not vary between the three sites; in 2010, its yearly average was 8 % at each site.

OM, EC, SIA, Na_Cl, and CM accounted for almost 81 % of the PM_2.5_ mass in Katowice, 74 % in Gdańsk, and 90 % in Diabla Góra. The annual average TM contribution to the PM_2.5_ mass was not greater than 0.2 % at each site.

The great differences in the PM_2.5_ concentrations between the winter and the summer, or the heating and the non-heating periods, and between the three sites in the concentrations of PM_2.5_-related OM + EC and in the PM_2.5_-related SIA, were due to the combustion of hard and brown coal for the energy production and to the meteorological conditions. The Na_Cl content of PM_2.5_ in Katowice, southern Poland, depended on combustion of fossil fuels, householg garbage, and biomass for heating in the cold part of the year (the dependence was weak in Diabla Góra). In Gdańsk, northern Poland, especially in the spring and autumn, sea spray might have affected the chemical composition of PM_2.5_.

The highest hazard from PM_2.5_ occurs in Katowice, southern Poland, in winter, when very high concentrations of PM_2.5_ and PM_2.5_-related carbonaceous matter, including BaP, are maintained by poor natural ventilation in cities, weather conditions, and the highest level of industrialization in Poland. In less industrialized northern Poland, where the aeration in cities is better, rather gaseous than solid fuels are used, the health hazard from ambient PM_2.5_ is much lower.

In Polish cities, where the air pollution is not uniformly distributed over the long, naturally distinguishable, heating or non-heating periods, an important factor for the reasoning about the PM_2.5_-related air pollutants is the selection of the period in the year. The air pollution in each short period of the year (spring, summer, autumn, winter) has some specific features that are ignored when averaging over the longer periods. Winter in Poland is the most critical period for air pollution and averaging the concentrations of PM_2.5_ and PM_2.5_-bound substances over a whole heating season neglects the more serious effects of winter alone. For example, the ionic balance examinations of PM_2.5_ showed the lack of NH_4_NO_3_ in secondary inorganic aerosol, and the probable acidity of PM_2.5_ in the big cities in the summer of 2010, which were not observed for the whole non-heating period. It seams that any proper approach to atmospheric aerosol in Poland cannot avoid the whole-year continuous observations with the analysis of the results in the respective periods (seasons) of the year. However, in the future, similar investigations of the chemical composition of PM should be based on daily sampling. Daily sampling enables more precise source apportionment with the use of commonly known and well described in the literature methods.
